# Identification and Validation of Ubiquitin-Specific Proteases as a Novel Prognostic Signature for Hepatocellular Carcinoma

**DOI:** 10.3389/fonc.2021.629327

**Published:** 2021-02-25

**Authors:** Wenkai Ni, Saiyan Bian, Mengqi Zhu, Qianqian Song, Jianping Zhang, Mingbing Xiao, Wenjie Zheng

**Affiliations:** ^1^Department of Gastroenterology, Affiliated Hospital of Nantong University, Nantong, China; ^2^Endoscopy Center and Endoscopy Research Institute, Zhongshan Hospital, Fudan University, Shanghai, China; ^3^Research Center of Clinical Medicine, Affiliated Hospital of Nantong University, Nantong, China; ^4^Department of Radiology, Wake Forest School of Medicine, One Medical Center Boulevard, Winston-Salem, NC, United States

**Keywords:** ubiquitin-specific proteases, hepatocellular carcinoma, prognosis, risk signature, molecular target

## Abstract

**Purpose:**

Ubiquitin-specific proteases (USPs), as a sub-family of deubiquitinating enzymes (DUBs), are responsible for the elimination of ubiquitin-triggered modification. USPs are recently correlated with various malignancies. However, the expression features and clinical significance of USPs have not been systematically investigated in hepatocellular carcinoma (HCC).

**Methods:**

Genomic alterations and expression profiles of USPs were investigated in CbioPortal and The Cancer Genome Atlas (TCGA) Liver hepatocellular carcinoma (LIHC) dataset. Cox regression and least absolute shrinkage and selection operator (LASSO) analyses were conducted to establish a risk signature for HCC prognosis in TCGA LIHC cohort. Subsequently, Kaplan-Meier analysis, receiver operating characteristic (ROC) curves and univariate/multivariate analyses were performed to evaluate the prognostic significance of the risk signature in TCGA LIHC and international cancer genome consortium (ICGC) cohorts. Furthermore, we explored the alterations of the signature genes during hepatocarcinogenesis and HCC progression in GSE89377. In addition, the expression feature of USP39 was further explored in HCC tissues by performing western blotting and immunohistochemistry.

**Results:**

Genomic alterations and overexpression of USPs were observed in HCC tissues. The consensus analysis indicated that the USPs-overexpressed sub-Cluster was correlated with aggressive characteristics and poor prognosis. Cox regression with LASSO algorithm identified a risk signature formed by eight USPs for HCC prognosis. High-risk group stratified by the signature score was correlated with advanced tumor stage and poor survival HCC patients in TCGA LIHC cohort. In addition, the 8-USPs based signature could also robustly predict overall survival of HCC patients in ICGC(LIRI-JP) cohort. Furthermore, gene sets enrichment analysis (GSEA) showed that the high-risk score was associated with tumor-related pathways. According to the observation in GSE89377, USP39 expression was dynamically increased with hepatocarcinogenesis and HCC progression. The overexpression of USP39 was further determined in a local HCC cohort and correlated with poor prognosis. The co-concurrence analysis suggested that USP39 might promote HCC by regulating cell-cycle- and proliferation- related genes.

**Conclusion:**

The current study provided a USPs-based signature, highlighting its robust prognostic significance and targeted value for HCC treatment.

## Introduction

Hepatocellular carcinoma is one of the most common malignancies worldwide with significant clinical, economic, and psychological burdens (1). Liver resection, ablation, and liver transplantation are potentially curative strategies for HCC patients at early stage, while a major proportion of HCC patients are diagnosed with intermediate and advanced stages with limited approaches (2). Currently, systemic therapy remains essential for advanced-stage HCC, including targeted agents and immune checkpoint inhibitors (3). However, HCC patients are generally inclined to poor prognosis with recurrence and chemoresistance. With the advancements in multi-omics profiling, recent studies have provided prognostic candidates for potential application of clinic. For the current status, it is of great significant to identifying robust molecular biomarkers to predict HCC patients’ outcome.

Ubiquitination (Ub) is one of the most common post-translational protein modifications that has been implicated in multiple biological processes, including embryonic development, cell cycle, and even oncogenesis (4). The dominant forms of Ub are recognized as mono-ubiquitination and Lys48/Lys63-linked polyubiquitination (5). The Ub processes are commonly mediated by E1-ubiquitin-activating enzymes, E2-ubiquitin-binding enzymes, E3-ubiquitin ligases, and deubiquitinating enzymes (DUBs) (6). DUBs, as proteolytic enzymes, are responsible for the elimination of ubiquitin-triggered modification, which could be further classified into eight sub-families such as ubiquitin-specific proteases (USPs), ubiquitin COOH terminal hydrolases (UCHs), Machado-Josephine domain-containing proteases (MJDs), ovarian tumor-associated proteases (OTUs), zinc finger–containing ubiquitin peptidases (ZUFSPs), and motif interacting with ubiquitin-containing novel DUB family (MINDY), Jab1/MPN domain-associated metallopeptidase (JAMM) domain proteins, and monocyte chemotactic protein-induced protein (MCPIP) (7). Of them, through deubiquitinating a wide range of substrates, USPs family members are involved in various physiological and pathological processes. Our previous study has suggested USP7 as a drug-able target that promoted chemoresistance of HCC (8). Recently, increasing studies indicate that USPs are implicated in the progression of HCC. USP22 could facilitate the hypoxia-induced stemness of HCC cells by regulating HIF1α/P53 signaling (9). USP5 enhanced epithelial-mesenchymal transition (EMT)-induced metastasis by stabilizing SLUG (10). Moreover, USP21 could promote the malignant transformation of the normal human hepatocytes and increased the tumorigenicity of the HCC cells by activating the ERK signaling through the stabilization of MEK2 (11). In contrast, USPs may also act as tumor suppressors in HCC progression. USP10 was reported to inhibit tumor growth and inactivate mTORC1/AKT signaling by stabilizing AMPKα and PTEN in HCC cells (12). Besides of regulating malignant behaviors, USPs were also considered as prognostic markers. Previous studies suggested that USP4, USP7, USP11, and USP33 were correlated with poor survival of HCC patients (13–16).

However, none integrated analysis of USPs has been performed for HCC till now. The current study systematically investigated the expression features and clinical significance of USP family members in HCC. Additionally, we established a USP family-based prognostic model from TCGA datasets and further validated it in ICGC (LIRI-JP) cohort. Considering the specific role of the USP family in HCC, we further explored the relationship between the signature genes and the landscape of HCC progression in GSE89377. Moreover, the expression features and clinical implications of USP39 were explored in a local HCC cohort.

## Methods and Materials

### Patients Information

HCC and adjacent tissues were obtained from 16 HCC patients who received hepatectomy at Affiliated Hospital of Nantong University (Nantong, Jiangsu, China) in 2018, which were frozen for western blotting. Liver specimens were collected from 106 patients with HCC underwent surgery at Affiliated Hospital of Nantong University between March 2012 and June 2017. Clinical information was recorded in detail, including each patient’ clinical parameters and post-surgery follow-up. All sections were pre-checked histologically. Written informed consent was obtained from each patient. This study was approved by the Ethics Committee of Affiliated Hospital of Nantong University.

### Data Acquisition and Preprocessing

The expression profiles of the 57 USPs in 374 HCC patients with clinical information was downloaded from TCGA LIHC datasets (https://cancergenome.nih.gov/) through the R package “TCGA-Assembler”. Heatmap was performed to visualize the expression levels of the USPs in HCC and normal tissues in TCGA LIHC cohort. The RNA-seq data of 232 HCC cases was also extracted from ICGC cohort (https://dcc.icgc.org/projects/LIRI-JP). The clinical parameters of the TCGA and ICGC cohort were shown in **Table 1**. The mRNA profiles of USPs in HCC-related stratified groups were extracted from GSE89377 and multiple GSE datasets.

**Table 1:** The clinical characteristic information of the HCC patients in TCGA and ICGC.

Characteristics	TCGA(%)	ICGC (%)
Number of Patients	374	232
**Age**		
<60	177 *(47.32)*	64*(27.59)*
≥60	196*(52.41)*	168*(72.41)*
NA	1*(0.27)*	NA
**Gender**		
Male	253*(67.65)*	171 *(73.71)*
Female	121*(32.35)*	61 *(26.29)*
**Survival status**		
Alive	238*(63.64)*	189*(81.47)*
Dead	130*(34.76)*	43 *(18.53)*
NA	6*(1.60)*	NA
**Stage**		
I	173*(46.26)*	36*(15.52)*
II	87*(23.26)*	106*(45.69)*
III	85*(22.73)*	71*(30.60)*
IV	5*(1.34)*	19*(8.19)*
NA	24*(6.42)*	NA
**Histological grade**		
G1	55*(14.71)*	NA
G2	178*(47.59)*	NA
G3	124*(33.16)*	NA
G4	12*(3.21)*	NA
NA	5*(1.34)*	NA
**T classification**		
T1	183*(48.93)*	NA
T2	95*(25.40)*	NA
T3	80*(21.39)*	NA
T4	13*(3.48)*	NA
NA	3*(0.80)*	NA
**N classification**		
N0	254*(67.91)*	NA
N1	4*(1.07)*	NA
NX	115*(30.75)*	NA
NA	1*(0.27)*	NA
**M classification**		
M0	268*(71.66)*	NA
M1	4*(1.07)*	NA
MX	102*(27.27)*	NA

NA, not available.

### PPI Network Construction, Correlation Analysis, and Consensus Clustering Analysis

An interaction network of 57 USPs was recapitulated by STRING (http://string-db.org). NetworkAnalyst (networkanalyst.ca) was used to predict the liver-specific protein-protein interaction (PPI) of the USPs. In addition, the Pearson correlation analysis was performed to calculate the associations among the 57 USPs. The LIHC cohort was stratified into sub-groups by consensus expression of USPs with “ConsensusClusterPlus” R package. Principal component analysis (PCA) was carried out with the “prcomp” function of the “stats” R package.

### Construction of Prognostic Signature

The correlation of USPs with clinical outcome of HCC patients was evaluated by Univariate Cox regression test. USPs with *P*<0.05 were further enrolled into Univariate Cox regression test by using the least absolute shrinkage and selection operator (LASSO) algorithm with the “glmnet” R package. An 8-gene prognostic signature was screened based on the minimum criteria. The risk score of each patient was calculated according to the normalized expression level of each gene and its coefficients. The formula was listed as follows: The coefficient (*gene_i_*) was derived from the Cox proportional hazards regression analysis. The TCGA LIHC cohort was divided into two sub-groups based on the median value of the risk score. t-SNE were performed to explore the distribution of different risk groups by using the “Rtsne” R package. In addition, to explore the potential function, gene ontology (GO) was performed based on the differential gene profiles of the two groups (|log_2_FC| ≥ 1, FDR <0.05).

### Evaluating the Prognostic Value of the Gene Signature

The correlation of risk score with clinicopathological features (age, gender, grade, stage, T, N, and M status) was evaluated by Chi-square test and visualized with heatmap. Kaplan-Meier analysis with log-rank test was conducted to compare the difference of overall survival between patients at high- or low- risk group. Stratified analysis was performed to evaluate the prognostic significance of the risk score in cases at different stages and grades. Receiver operating characteristic (ROC) curve was conducted to assess the predictive performance of the signature model. Univariate and Multivariate Cox regression analyses were performed to define the risk score as an independent prognosis predictor for HCC patients in the TCGA cohort. To validate the prognostic value of the USPs-based signature, the Kaplan-Meier analysis, ROC analysis, Univariate and Multivariate Cox regression analyses were also performed in ICGC cohort.

### Gene Sets Enrichment Analysis

To identify enriched pathways associated with the signature, gene set enrichment analysis (GSEA) was performed on the high-risk sub-groups of the TCGA and ICGC cohorts, respectively. The analysis was based on GSEA v.3.0. Molecular Signatures Database v.7.0. Gene sets with *P* value < 0.05 and FDR < 25% were considered significantly enriched.

### Expression Profiles and Functional Prediction for USP39

The mRNA profiles of USP39 in Pan-cancers and the correlation of USP39 with proliferation and cell cycle-related genes was analyzed by TIMER database (cistrome.shinyapps.io/timer). The significantly correlated genes with USP39 in TCGA LIHC dataset and corresponding GSEA were analyzed by LinkedOmics (www.linkedomics.org). The mRNA profiles of USP39 in HCC cases at different stages and grades were obtained from Ualcan (ualcan.path.uab.edu).

### Immunohistochemistry (IHC) and Evaluation

The sections of HCC tissues were deparaffinized in xylene, dehydrated in gradient concentrations of ethanol. After incubated in sodium citrate buffer for antigen retrieval, the slides were blocked in BSA for 1 h at room temperature. Then the samples were sequentially incubated with the indicated primary antibody (USP39, 1:200, Santa Cruz, USA) and secondary antibody. At last, the sections were visualized by 3,3′-diaminobenzidine (DAB, Kem-En-Tec Diagnostics, Denmark). Staining of USP39 was independently scored by two pathologists. The statistical analysis of the IHC results were performed as previously described (17). The IHC score was calculated by combining staining intensity and positive percentages. The positive percentages were scored as follows: 0 (0%); 1(1–10%), 2 (11–50%), 3 (51–80%), and 4 (≥81%). The staining intensity was scored as 0 (negative), 1 (weak), 2 (moderate), and 3 (strong). The final score was calculated by multiplying the percentage score with the intensity score. Score of 4-12 was considered high expression of USP39, while score less than 4 was defined as low expression.

### Western Blotting

The total protein of each sample was extracted by using radioimmunoprecipitation assay buffer (RIPA) and separated by a sodium dodecyl sulfate (SDS) gel. Following transferred onto polyvinylidene difluoride (PVDF) membranes, the samples were subsequently blocked in 5% BSA for 2 h and incubated in primary antibody (USP39, Santa, USA; GAPDH, Abcam, USA) at a concentration of 1:1,000 overnight. Then the membranes were rinsed in TBST for three times, followed by exposing to horseradish peroxidase (HPR) -conjugated secondary antibodies (Abcam, USA) for 2 h at room temperature. Ultimately, the intensity of the membranes was detected by using the enhanced chemiluminescence (ECL) kit (Millipore, USA).

### Statistical Analyses

The statistics in this study were performed by using R software (Version 3.5), GraphPad Prism software (Version 7.0), and SPSS (Version 23.0). The survival difference of overall survival between two groups was compared by Kaplan-Meier analysis with a log-rank test. Univariate and multivariate analyses were conducted by using the Cox proportional hazards regression model. The chi-square test was used to evaluate the relationship between the risk score and clinicopathological variables. One-way ANOVA and multiple comparison were used to determine the differential expression of USPs among sub-groups in GSE89377. *P* < 0.05 was considered as statistically significant.

## Results

### Genomic Alterations of the USP Family in TCGA LIHC Cohort

**Figure 1:**
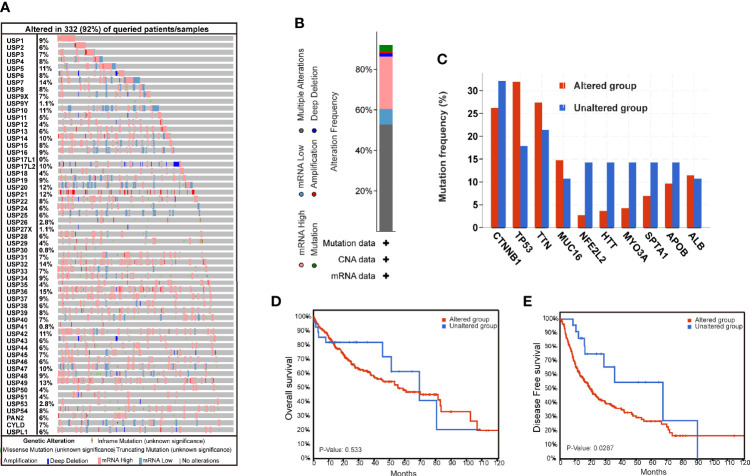
The genomic alterations of ubiquitin-specific proteases in HCC tissues. **(A)** The Oncoprint of 57 ubiquitin-specific proteases (USPs) in TCGA LIHC dataset by CbioPortal. **(B)** Integrated analysis of the USPs genomic alteration proportion in LIHC dataset. **(C)** The top mutated genes in the USPs-altered group and USPs-unaltered groups. **(D, E)** The Kaplan-Meier curves of the overall survival and disease-free survival for the HCC patients in USPs-altered group and USPs-unaltered groups.

The genomic alterations of USPs are presented in **Figure 1**. A total of 57 USPs were involved in this study. According to the OncoPrint analyzed by CbioPortal, USP36 (15%), USP7 (14%), and USP32(14%) were three top-altered genes (**Figure 1A**). Generally, USP family members altered in 91.8% of the whole HCC cases (**Figure 1B**). Of them, the frequency of mutation, amplification, deep deletion, mRNA high, mRNA low, and multiple alteration was 3.44%, 0.57%, 1.72%, 25.78%, 7.74%, and 52.72%, respectively. As shown in **Figure 1C**, five well-known HCC-related genes had higher alteration frequency in the USPs-altered group. Furthermore, the altered group showed poorer disease-free survival (DFS, *P*=0.0287), though the difference of the overall survival was not statistically significant (**Figure 1D, E**).

### The Expression Features of USPs in HCC

**Figure 2:**
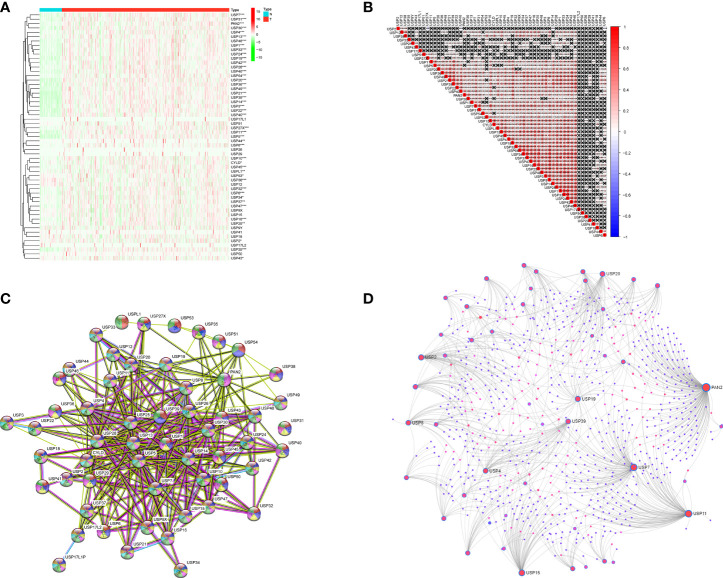
The expression features and interactions of ubiquitin-specific proteases family in HCC. **(A)** The expression levels of 57 USPs in HCC tissues and normal liver tissues evaluated in TCGA datasets. **(B)** Spearman correlation analyses of the 57 USPs in LIHC cohort. **(C)** The interactions among the 57 USPs was analyzed by STRING. **(D)** Liver specific interaction of the 57 USPs was predicted by Networkanalyst. ***P < 0.001; **P < 0.01; *P < 0.05.

The expression profiles of USPs in 374 HCC tissues and 50 normal liver tissue were extracted from TCGA LIHC cohort. Compared with normal liver tissues, a majority of USPs (45/57) presented higher expression in HCC tissues (**Figure 2A**). As shown in **Figure 2B**, the correlation analysis showed that the most relevance among all the USPs was observed in USP34/USP37 (*r* = 0.79) and USP1/USP24 (*r* = 0.79). Furthermore, we used STRING to establish the interactive network among the 57 USPs, in which USP1, USP39, USP5, USP13, and USP25 seemed to be the hub genes (**Figure 2C**). In addition, NetworkAnalyst (networkanalyst.ca) was used to predict the liver-specific protein-protein interaction (PPI) of the 57 USPs (**Figure 2D**). GO and KEGG analyses further indicated that the nodules in this PPI network were enriched in biological processes like cellular protein catabolic process, and protein ubiquitination, as well as pathways like cell cycle, necroptosis, viral carcinogenesis, Hippo signaling pathway, and NF-kappa B signaling pathway (**Table 2**).

**Table 2:** GO_BP and KEGG analyses of the liver-specific interaction of USPs.

Pathway	Total	Expected	Hits	P Value	FDR
**GO_BP analysis**					
Cellular protein catabolic process	518	25.6	114	5.77E-44	4.73E-41
Protein catabolic process	644	31.8	121	3.12E-39	1.28E-36
Cellular macromolecule catabolic process	849	42	138	1.44E-37	3.92E-35
Macromolecule catabolic process	1070	52.9	148	4.12E-32	6.76E-30
Proteolysis	1100	54.1	133	3.10E-23	4.24E-21
Protein modification	713	35.2	96	8.08E-20	9.47E-18
Protein ubiquitination	658	32.5	88	5.17E-18	5.30E-16
Cellular catabolic process	2140	106	189	7.86E-17	7.16E-15
Catabolic process	2560	127	205	8.42E-14	6.90E-12
Interphase of mitotic cell cycle	435	21.5	61	1.14E-13	8.47E-12
**KEGG analysis**					
Ubiquitin mediated proteolysis	137	8.02	32	7.18E-12	2.28E-09
Cell cycle	124	7.26	29	6.92E-11	1.10E-08
Necroptosis	162	9.49	31	3.13E-09	3.32E-07
Endocytosis	244	14.3	39	6.46E-09	5.14E-07
Epstein-Barr virus infection	201	11.8	32	1.71E-07	9.76E-06
Oocyte meiosis	125	7.32	24	1.84E-07	9.76E-06
Viral carcinogenesis	201	11.8	30	1.72E-06	7.80E-05
Hippo signaling pathway	154	9.02	25	2.74E-06	0.000109
Pathways in cancer	530	31	57	3.97E-06	0.000133
NF-kappa B signaling pathway	100	5.85	19	4.17E-06	0.000133

### Consensus Clustering of the USPs in TCGA LIHC Cohort

**Figure 3:**
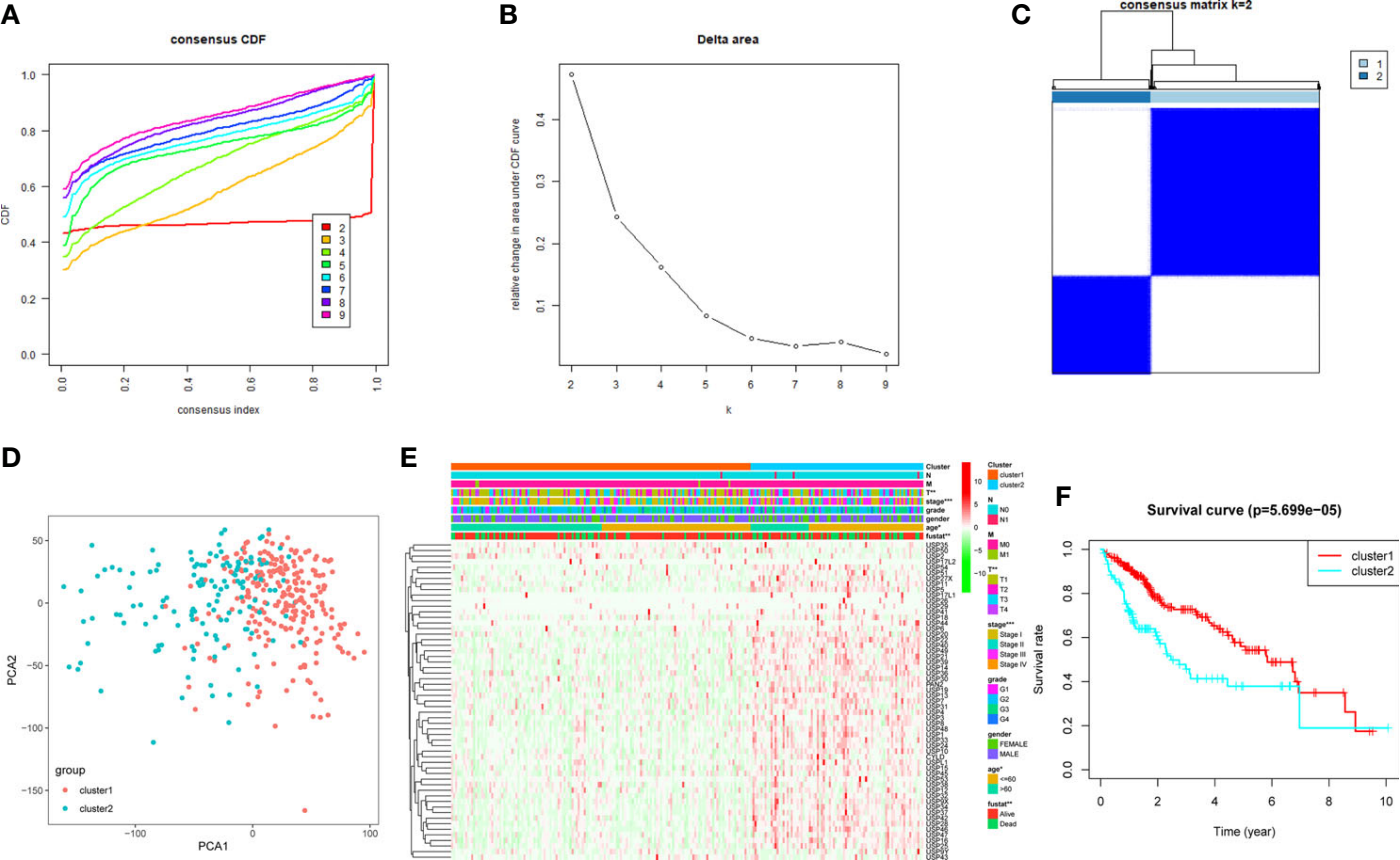
Consensus clusters of the USPs. **(A, B)** Consensus clustering model with cumulative distribution function (CDF) with k from 2 to 9; **(C)** The LIHC cohort stratified into two clusters (k = 2); **(D)** principal component analysis (PCA) of the total mRNA profiles of the two clusters; **(E)** Heatmap indicated the correlation of cluster 2 with clinicopathologic parameters. **(F)** The Kaplan-Meier curves of the overall survival of HCC patients in the two clusters. ***P < 0.001; **P < 0.01; *P < 0.05.

Based on the expression features of USPs and CDF value, 374 HCC samples of TCGA cohort were stratified into two clusters by using the Consensus ClusterPlus package (k = 2, **Figures 3A–C**). Furthermore, the PCA showed that the two clusters could be well- distinguished in the whole TCGA LIHC cohort (**Figure 3D**). Then, we further explored the association between the USPs-based clusters and the clinicopathological parameters of HCC patients in LIHC cohort. As the heatmap illustrated in **Figure 3E**, USPs-overexpressed cluster 2 was significantly correlated with age, neoplasm stage, tumor growth, and survival status. Consistently, the patients at cluster 2 with USPs overexpression had poorer overall survival than cases of cluster 1 (**Figure 3F**, *P*<0.001), suggesting the prognostic potential of USP family for HCC patients.

### Construction of the USPs-Based Signature in TCGA LIHC Cohort

**Figure 4:**
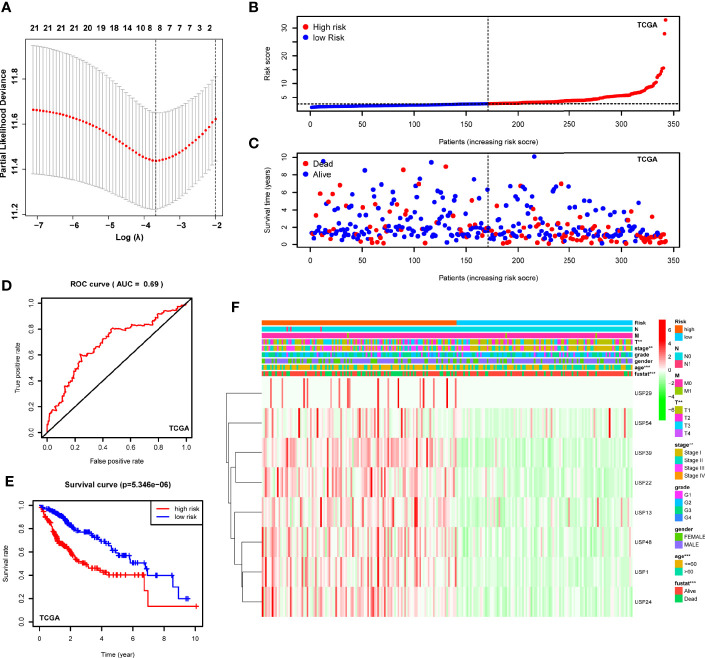
Construction of the USPs-based signature in TCGA LIHC cohort. **(A)** The coefficients of the 8-gene signature were calculated by multivariate Cox analysis with LASSO. **(B)** The distribution and median value of the risk scores in the TCGA LIHC cohort. **(C)** The distributions of OS status and risk score in the TCGA LIHC cohort. **(D)** The ROC curve was calculated to evaluate the predictive efficiency of the USPs-based signature in TCGA. **(E)** The Kaplan–Meier curves of overall survival for HCC patients at high-risk group and low-risk group in TCGA. **(F)** The correlation of the high or low risk score with clinicopathologic parameters in the TCGA LIHC cohort. ROC, receiver operator curve. ***P < 0.001; **P < 0.01.

With the implications in HCC prognosis, we further conducted the univariate Cox regression analyses to evaluate the prognostic significance of USPs. As listed in **Supplementary Table 1**, 21 USPs were significantly correlated with poor overall survival of the HCC patients, including USP1, USP10, USP11, USP13, USP14, USP15, USP19, USP21, USP22, USP24, USP28, USP29, USP32, USP33, USP36, USP37, USP39, USP42, USP46, USP48, and USP54. Next, the 21 USPs were enrolled into the Cox proportional hazards regression analysis with LASSO algorithm (**Figure 4A**). Eight genes, including USP1, USP13, USP22, USP24, USP29, USP39, USP48, and USP54, were ultimately screened to establish the signature based on the minimum criteria. According to the expression level of the USPs and coefficients, we stratified the TCGA LIHC cohort into a high-risk group and a low-risk group based on the median risk score. t-SNE analysis indicated the efficiency to distinguish different risk group (**Figure 4B**). As shown in **Figure 4C**, patients at high-risk group had a probability of poor survival than that in low-risk group. Next, the ROC analysis suggested that the risk signature could robustly predict OS for HCC patients in TCGA LIHC cohort (AUC = 0.69, **Figure 4D**). In addition, Kaplan-Meier analysis demonstrated that HCC patients with high-risk score had shorter overall survival compared to the cases with low-risk score (*P*<0.001, **Figure 4E**). Then we analyzed the correlation of the risk signature with clinical parameters in TCGA cohort. High-risk group was significantly correlated with aggressive phenotypes such as tumor size, neoplasm stage, and survival status (**Figure 4F**). Additionally, we conducted stratified analysis in the sub-groups of TCGA LIHC cohort. For HCC patients at stage I and II, high-risk score led to a poorer overall survival (**Supplementary Figure 1A**, *P*<0.001). However, though the general survival time was obviously shorter in high-risk group, the difference was not statistically significant for patients at stage III and IV (**Supplementary Figure 1B**, *P*=0.103). In contrast, patients at high-risk score displayed a significantly poorer OS in sub-groups of grade I&II (**Supplementary Figure 1C**, *P*<0.001) or grade III & IV (**Supplementary Figure 1D**, *P*=0.026).

### Validating the Signature in ICGC Cohort

**Figure 5:**
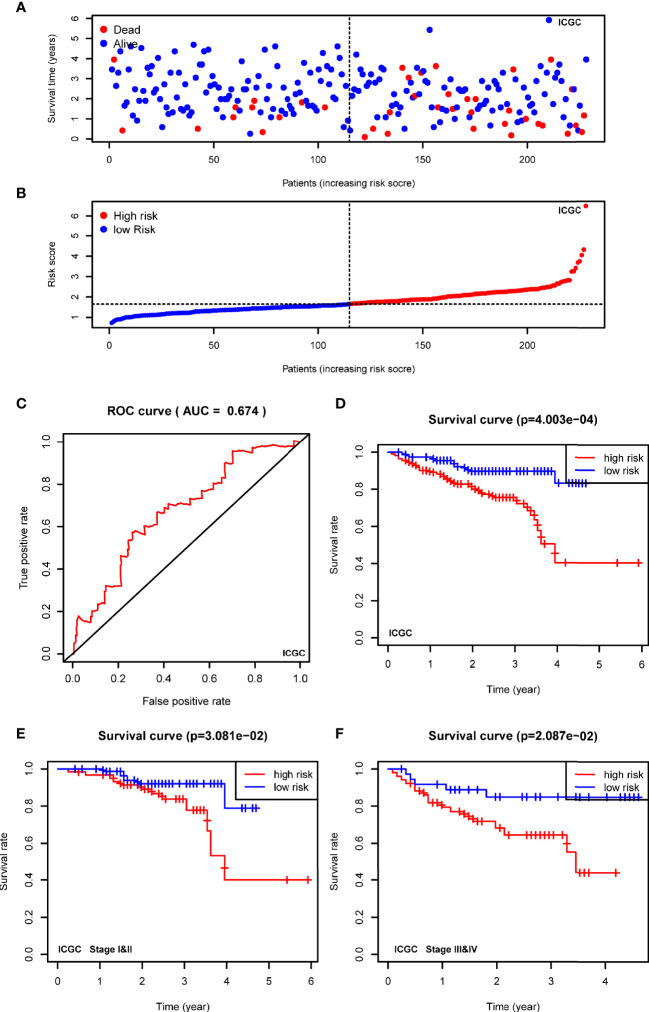
Validating the prognostic value of the USPs-based signature in ICGC cohort. **(A)** The distribution and median value of the risk scores in the ICGC cohort. **(B)** The distributions of OS status and risk score in the ICGC cohort. **(C)** The ROC curve was calculated to evaluate the predictive efficiency of the USPs-based signature in ICGC cohort. **(D)** The Kaplan–Meier curves of overall survival for HCC patients at high-risk group and low-risk group in ICGC cohort. **(E, F)** The Kaplan–Meier curves of HCC patients at stage I&II and stage III&IV in ICGC cohort. ICGC, International Cancer Genome Consortium.

To confirm the robustness of the signature, we further evaluated the risk model in ICGC cohort (LIRI-JP). The patients from the ICGC cohort were also categorized into two groups according to the median risk score calculated by the formula established in the TCGA cohort. The t-SNE analysis demonstrated that patients in two high- and low-groups were distributed in discrete dots (**Figure 5A**). In consistent with the results of TCGA, the cases in the high-risk group had a probability of poorer survival (**Figure 5B**). The ROC analysis also confirmed the predictive performance of the risk model in ICGC cohort (**Figure 5C**, AUC=0.674). In addition, Kaplan-Meier analyses indicated that HCC patients with high-risk score had reduced overall survival time (**Figure 5D**, *P*<0.001). As shown in **Figures 5E, F**, the stratified analysis showed that high-risk score was correlated with shorter OS in cases both of I&II stages (**Figure 5E**, *P*=0.0308) and III&IV stages (**Figure 5F**, *P*=0.0209) in the ICGC cohort.

### Identify the USPs-Based Signature as an Independent Prognostic Factor of HCC

**Figure 6:**
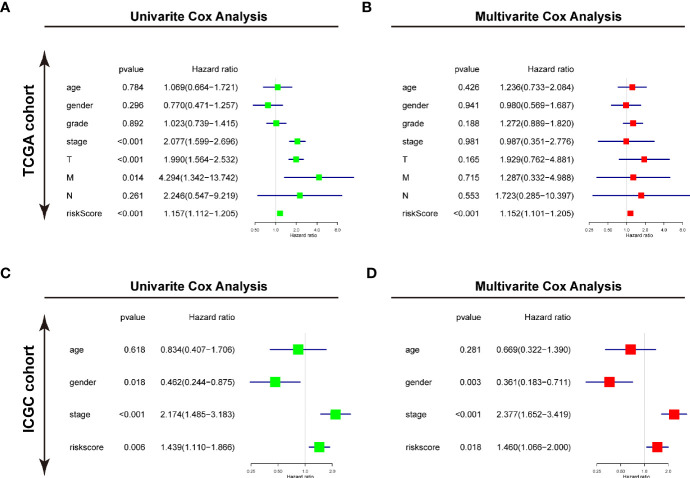
Identifying the USPs-based signature as an independent factor for HCC prognosis. **(A)** Univariate Cox analyses of the clinicopathological factors (including the risk score) and overall survival in the TCGA LIHC cohort. **(B)** Multivariate Cox analyses of the clinicopathological factors (including the risk score) and overall survival in the TCGA LIHC cohort. **(C)** Univariate Cox analyses in the ICGC cohort. **(D)** Multivariate Cox analyses in the ICGC cohort. ICGC, International Cancer Genome Consortium.

Furthermore, the univariate and multivariate Cox regression analyses were conducted to evaluate the risk signature as an independent prognostic factor in the two cohorts. For TCGA cohort, the univariate Cox analysis demonstrated that the risk score (*P*<0.001, HR = 1.157, 95% CI = 1.112–1.205), neoplasm stage, T status, and M status were potential hazard factors (**Figure 6A**). Further multivariate Cox regression analysis elucidated that the risk score was an independent factor (*P*<0.001, HR = 1.152, 95% CI = 1.101–1.205) of HCC (**Figure 6B**). For ICGC cohort, the univariate Cox analysis the risk score (*P*=0.006, HR = 1.439, 95% CI = 1.110–1.866) and neoplasm stage were candidate factors (**Figure 6C**). In accordance with the observation in TCGA cohort, the risk score was also recommended as an independent predictor for OS by the multivariate Cox regression analysis in the ICGC cohort (**Figure 6D**, *P*=0.018, HR = 1.460, 95% CI = 1.066–2.000).

### Functions and Pathways Correlated With the USPs-Based Signature

**Figure 7:**
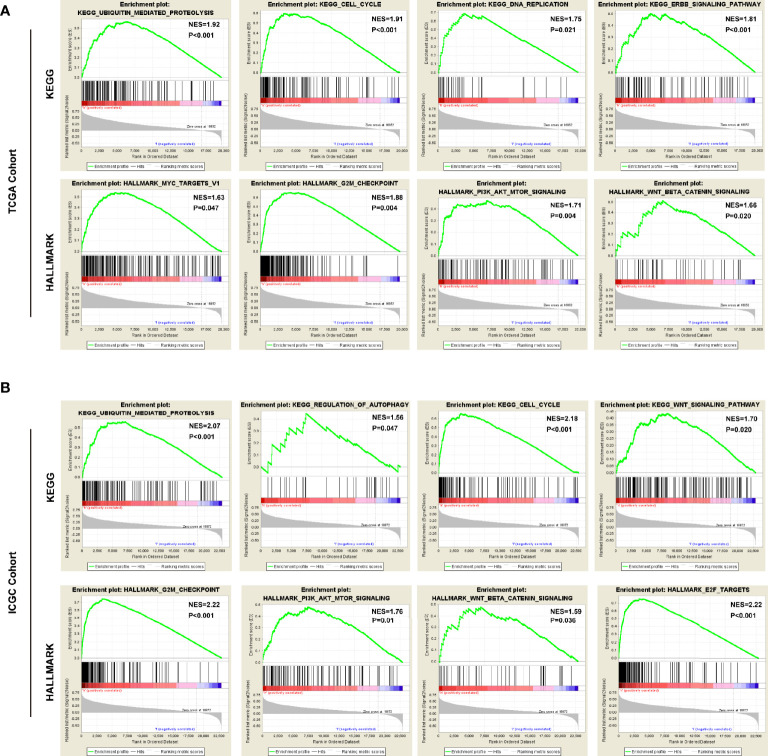
The pathways correlated with the high-risk score. The gene set enrichment analysis (GSEA) was performed in the TCGA and ICGC cohorts to explore mechanisms underlying the 8-USPs based signature. **(A)** Four representative KGEE pathways and hallmarks in the high–risk group of TCGA cohort; **(B)** Four representative KGEE pathways and hallmarks in the high–risk group of ICGC cohort. NES, Normalized enrichment score.

To predict the biological functions, we conducted GO analyses in differential expression genes (DEGs) between high-risk and low-risk groups in both of TCGA cohort and ICGC cohort. the DEGs were enriched in biological process like DNA replication, nuclear division, ECM constituent, small molecule catabolic process, collagen-containing ECM, and peptidase inhibitor activity (**Supplementary Figures 2A, B**). Furthermore, we conducted GSEA with KEGG and Hallmarks to unravel the molecular mechanisms underlying the USPs-based signature **(****Supplementary Table 2****)**. As shown in **Figure 7A**, the ubiquitin mediated proteolysis, cell cycle, DNA replication, ERBB signaling, MYC targets, G2/M checkpoints, PI3K/AKT/mTOR signaling, and Wnt/β-catenin pathway were enriched in the high-risk group of the TCGA cohort. For the ICGC cohort, in addition to the pathways mentioned above, the significantly enriched pathways also included regulation of autophagy and E2F targets (**Figure 7B**).

### Expression Features of the Risk Genes in HCC Progression

**Figure 8:**
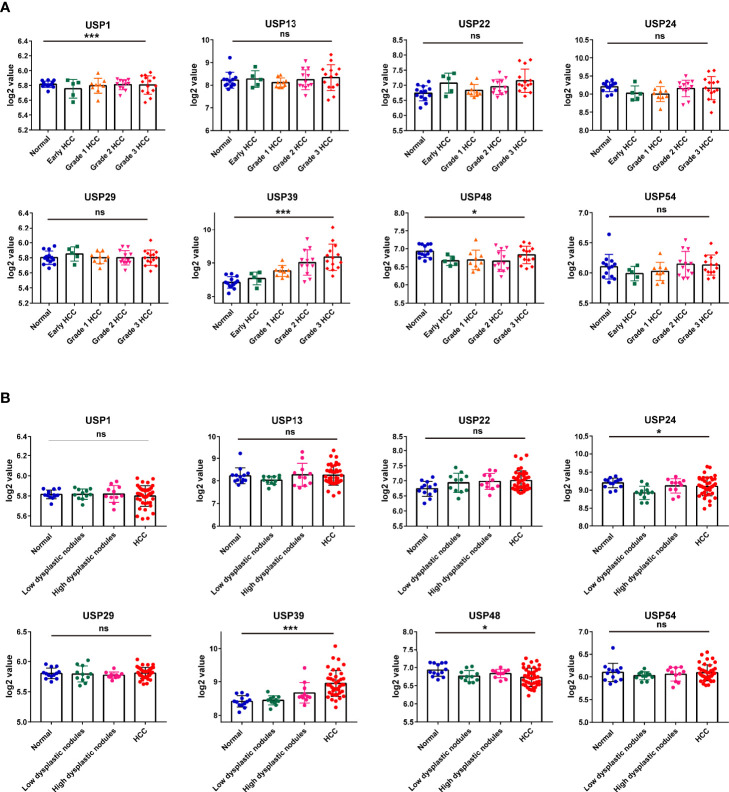
The expression features of the 8 risk genes in hepatocarcinogenesis and HCC cases at different grades in GSE89377 cohort. **(A)** The expression features of the eight USPs in cases at different tumor grades in GSE89377 cohort. **(B)** The expression features of the eight USPs in patients with dysplastic nodules and HCC in GSE89377 cohort. *P < 0.05; ***P < 0.001; ns, non-significance.

Then, we extracted the mRNA profiles from GSE89377 to analyze the expression of the 8 USPs in the HCC progression. At first, we compared the expression of the USPs in HCC staging, in which only USP39 presented the dynamically increasing characteristics (**Figure 8A****)**. It was consistent with the observation in Ualcan databases, by which USP39 expression was significantly upregulated in advanced stages and grades of HCC patients **(****Supplementary Figures 3A, B**). Interestingly, among the eight signature genes, USP39 was found elevated from normal control, dysplastic nodules with low grade, dysplastic nodules with high grade, to HCC cases, suggesting an enhanced expression tendency in hepatocarcinogenesis (**Figure 8B****)**. The observation above suggested the potential role of USP39 in HCC progression and hepatocarcinogenesis.

### The Expression Feature of USP39 in HCC

**Figure 9:**
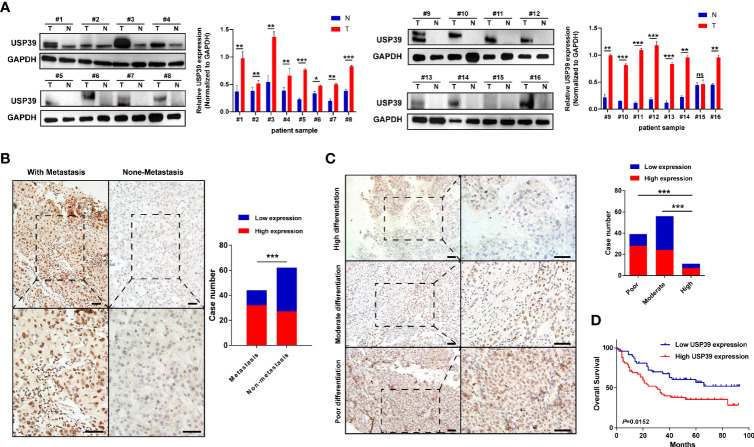
The protein expression features and prognostic significance of USP39 in HCC tissues. **(A)** The expression of USP39 in 16 pairs of HCC and adjacent tissues analyzed by western blotting. **(B)** Representative immunostaining images of USP39 in HCC cases with or without metastasis. **(C)** Representative immunostaining images of USP39 in HCC cases with high, moderate, or poor differentiation. **(D)** The Kaplan–Meier curves of HCC patients with high or low USP39 expression identified by immunohistochemistry. T, tumor tissues; N, adjacent tissues; ns, non-significance. ***P < 0.001; **P < 0.01; *P < 0.05.

Based on the observation in GSE89377 dataset, we further explored the potential functions and mechanisms regulated by USP39 (**Supplementary Table 3**). According to the pan-cancers analysis in TIMER dataset, the most significant difference of USP39 between pan-cancers and normal tissues was observed in HCC (**Supplementary Figure 4A**). In a serials of mRNA expression datasets, most of them (10/11) confirmed the overexpression of USP39 in HCC tissues (**Supplementary Figure 4B****)**. In protein level, as shown in **Figure 9A**, significantly elevated expression of USP39 was determined in 15/16 HCC tissues compared with the self-paired adjacent tissues analyzed by western blotting. Then we further explored the expression feature of USP39 in a local cohort including 106 HCC tissues by performing immunohistochemistry. USP39 was mainly distributed in the nucleus of HCC tissues. HCC cases with metastasis presented higher staining intensity of USP39 than the cases without metastasis (**Figure 9B**). Additionally, higher expression of USP39 was detected in poorly differentiated tissues than well differentiated HCC cases (**Figure 9C**). Furthermore, Kaplan-Meier analyses suggested that HCC patients with higher USP39 expression might have shorter overall survival (**Figure 9D**).

### The Clinical Implications and Mechanisms of USP39 in HCC

**Figure 10:**
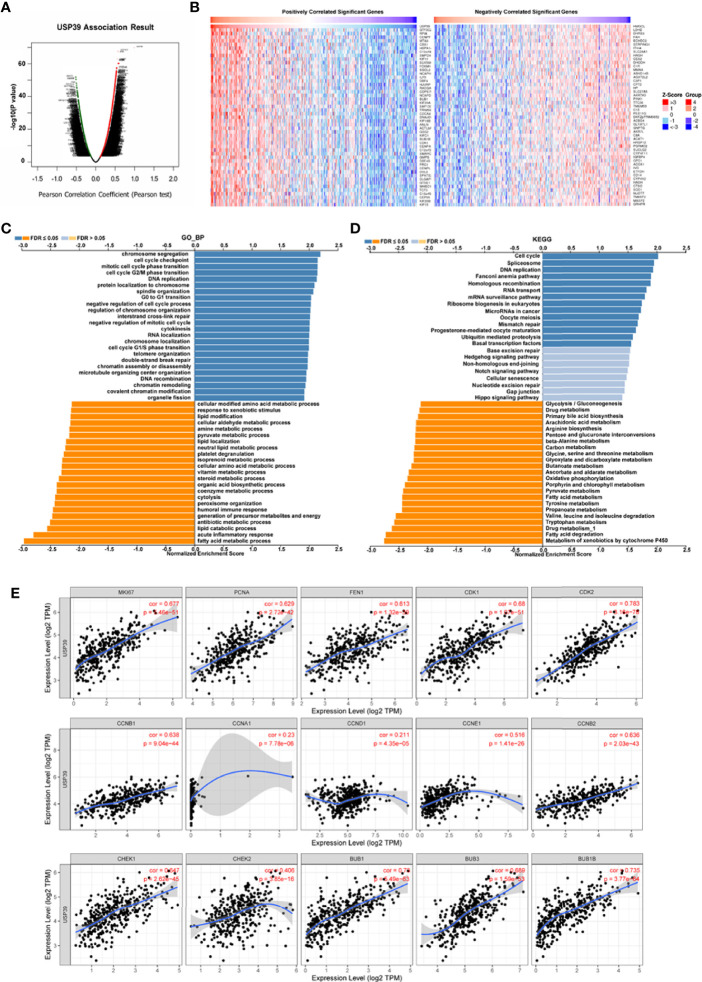
The potential function of USP39 in HCC. **(A, B)** The significantly positively- or negatively- correlated genes with USP39 in the TCGA LIHC cohort. **(C, D)** The USP39-regulated functions and pathways were calculated by GSEA with GO analysis and KEGG analysis. **(E)** The correlation of USP39 with cell cycle and proliferation-related genes in TCGA LIHC cohort were calculated by TIMER.

For the clinical investigation, USP39 overexpression was correlated with neoplasm stage, histological grade, and tumor size of HCC patients in LIHC cohort (**Table 3**). To further explore the biological functions mediated by USP39, we used LinkedOmics to examine USP39 co-expression mode in TCGA LIHC cohort (**Figure 10A**). The top 50 genes significantly correlated with USP39 in TCGA were elucidated in the heatmap (**Figure 10B**). GO term annotation by GSEA indicated that USP39 co-expressed genes were enriched in processes like cell cycle checkpoint, G0/G1 and G2/M transition, DNA replication, and cytokines (**Figure 10C**). The KEGG analysis showed the enrichment in cell cycle, DNA replication, and ubiquitin mediated proteolysis. Based on these, we analyzed the correlation of USP39 with cell cycle and proliferation-related genes in TCGA LIHC cohort (**Figure 10D**). As elucidated in **Figure 10E**, USP39 was significantly correlated with PCNA, FEN1, MKI67, CDK1/2, Cyclin B1/2, CHEK1/2, and BUB1/1B/3.

**Table 3:** Correlation of USP39 expression with clinical parameters of HCC patients in TCGA.

Clinical characteristics	Total(N)	Odds ratio in USP39	P value
Age (≥65 vs.<65)	370	1.54 (1.01,2.33)	0.044
Gender (female vs. male)	371	0.72 (0.47,1.11)	0.14
Stage (I/II vs. III/IV)	347	1.88 (1.15,3.07)	1.10E-02
Histological grade (G1/G2 vs. G3/G4)	366	1.8 (1.17,2.77)	7.00E-03
T (T1/T2 vs. T3/T4)	368	1.71 (1.06,2.76)	2.60E-02
N (N0 vs. N1)	256	2.73 (0.28,26.57)	0.356
M (M0 vs. M1)	270	2.87 (0.29,27.92)	0.331
Survival status (alive vs. dead)	371	0.65 (0.42,0.99)	0.046

## Discussion

Ubiquitination is a critical post-translational mechanism that plays multifaceted roles in multiple biological processes like apoptosis, cell-cycle progression, inflammatory responses, and transcriptional activities (18, 19). DUBs can inactivate the Ub signal from target proteins by trimming Ub chains (20). However, dysregulation of the DUBs may induce the malfunction of the ubiquitin system, which could subsequently regulate a serial of oncogenes or tumor suppressor genes (21, 22). A growing body of evidence suggests that USP sub-family is implicated in various malignancies (23). In the current study, we focused on the expression features and prognostic value of USPs for HCC. According to the hepatic expression levels in TCGA cohort, most of the USPs were overexpressed in HCC tissues. As elucidated in the liver-specific PPI, the nodules in this network were enriched in tumor or inflammation-related processes and pathways. Of them, Hippo signaling pathway and NF-kappa B signaling pathway have been frequently implicated in the genesis and progression of HCC.

Furthermore, the Consensus cluster analysis further indicated the correlation of USPs-enriched sub-cluster with neoplasm stage, tumor growth, and overall survival, suggesting the prognostic potential of the USP family for HCC patients. Then we screened the USPs with prognostic value evaluated in TCGA LIHC cohort, which were subsequently enrolled into multivariate analysis with LASSO algorithm. Ultimately, USP1, USP13, USP22, USP24, USP29, USP39, USP48, and USP54 were used to establish the risk signature. As shown in the survival analyses, the signature-derived risk score could robustly predict the overall survival in the entire TCGA LIHC cohort and sub-groups stratified by different stages and grades. To further confirm the prognostic value of the signature, we chose another HCC cohort ICGC (LIRI-JP). In consistence with the observation in TCGA cohort, it also had excellent performance in predicting the overall survival of HCC patients in the ICGC cohort. Remarkably, the univariate and multivariate Cox analyses in the two cohorts draw a consistent result that the USPs-based signature-derived risk score was an independent factor for the prognosis of HCC patients. For the potential mechanisms modulated by the USPs-based signature, we conducted the GO analysis on the DEGs and GSEA in the two cohorts. Interestingly, some well-known HCC-related pathways were correlated with the high-risk score, including cell cycle, DNA replication, ERBB signaling, MYC signaling, G2/M checkpoints, PI3K/AKT/mTOR signaling, Wnt/β-catenin pathway, autophagy, and E2F signaling. It was speculated that the activation in these tumorigenesis pathways might contribute to the poor survival of the patients with high-risk score.

Hepatocarcinogenesis is known as a multi-center and multi-step process, in which USPs played crucial roles (24, 25). Then GSE89377, a dataset with different HCC- related sub-groups, was used to investigate the expression characteristics of the USPs in HCC staging and hepatocarcinogenesis. For the eight signature USPs, only USP39 presented significantly enhanced expression with the advancing of histological grade. Subsequently, we also explored the expression levels of the eight genes in normal, dysplastic nodes (low and high grade), and HCC tissues. In accordance with the data in cases at different grades, USP39 displayed a dynamic increasing from pre-HCC status to HCC. However, USP13, USP22, and USP39, previously correlated with the malignant phenotypes of HCC cells (26–28), showed less differences among the sub-groups. It could be speculated that these USPs might be not key driver genes for HCC progression though they enhance aggressive behaviors of HCC cells. Instead, based on the observation in this GSE dataset, USP39 might serve as a hub gene that participates in tumorigenesis and HCC progression.

We further focused on exploring the clinical significance and potential function of USP39 in HCC. USP39 is a cysteine deubiquitinating enzyme belonging to the USP family. Overexpression of USP39 was observed in approximately half of the pan-cancers in TCGA dataset, of which HCC displayed the most differences between normal and tumor tissues. In addition, combining analyses of multiple datasets and experimental detection further confirmed the overexpression of USP39 in HCC tissues. Interestingly, the expression of USP39 also increased from pre-cancerous stage to HCC, which was consistent with the observation above and further suggested the implications of USP39 in hepatocarcinogenesis. Currently, the clinical significance of USP39 has not been investigated in HCC. Herein, we found that overexpression of USP39 was significantly correlated with neoplasm stage, histological grade, and tumor size. Through the co-concurrence analysis, we found that USP39 might be involved in cell cycle and DNA replication pathways. As is known, the proteins such as Cyclin family, CDKs, and checkpoint molecules are highly important to ensure proper proliferation, while deregulation of these proteins can result in various types of tumors. As expected, according to the analysis in TIMER, USP39 was significantly correlated with cell cycle- and proliferation- related genes. Accordantly, a previous study suggested the tumor-promotive role of USP39 in HCC cell lines. USP39 knockdown has been found to inhibit the proliferation and colony formation through downregulating the transcription factor Forkhead Box M1 (29). The observation above suggested that USP39 might be a potential molecular target for HCC treatment.

Despite of the encouraging performance of the USPs-based signature, there were certain limitations for the current study. The establishment and validation of the signature were based on the public sequence data. Further validation, such as prospective studies and clinical trials of HCC patients in multi-centers, might make the signature more convincing. In addition, the current study preliminarily predicted the functions and pathways modulated by USPs. The exact biological roles and mechanisms of UPSs in HCC remained to be investigated by more experimental assays.

## Conclusion

In conclusion, the current study investigated the expression features and potential functions of USPs in HCC. An 8-USPs-formed signature could robustly predict the prognosis of HCC patients in TCGA LIHC cohort and ICGC (LIRI-JP) cohort. In addition, USP39, one of the eight signature genes, might be a potential molecular target for hepatocarcinogenesis and HCC progression. Our study provided evidence for the future investigation into USP family in the prognostic significance and targeted value for HCC treatment.

## Data Availability Statement

The datasets presented in this study can be found in online repositories. The names of the repository/repositories and accession number(s) can be found in the article/**Supplementary Material**.

## Ethics Statement

The studies involving human participants were reviewed and approved by the Ethics Committee of Affiliated Hospital of Nantong University. The patients/participants provided their written informed consent to participate in this study.

## Author Contributions

WZ and MX conceived and designed the study. QS, WN, and JZ analyzed the data. SB and MZ drafted the paper. WZ revised the manuscript. All authors contributed to the article and approved the submitted version.

## Funding

This study was supported by grants from the National Natural Science Foundation (82070622, 81702419), the Key Research and Development Plan of Jiangsu Province (BE2020668, BE2019692), and the Nantong Science and Technology Project (MS12019013, MS12020020, MS22020005).

## Conflict of Interest

The authors declare that the research was conducted in the absence of any commercial or financial relationships that could be construed as a potential conflict of interest.
