# Supplementary material for: Identification and Validation of Ubiquitin-Specific Proteases as a Novel Prognostic Signature for Hepatocellular Carcinoma
Source: Front Oncol. 2021 Feb 25;11:629327. doi: 10.3389/fonc.2021.629327 (PMC7949004; doi:10.3389/fonc.2021.629327)
Supplement: Supplementary file 1 [file Presentation_1.pdf]

Supplementary Material

1.1 Supplementary Figures

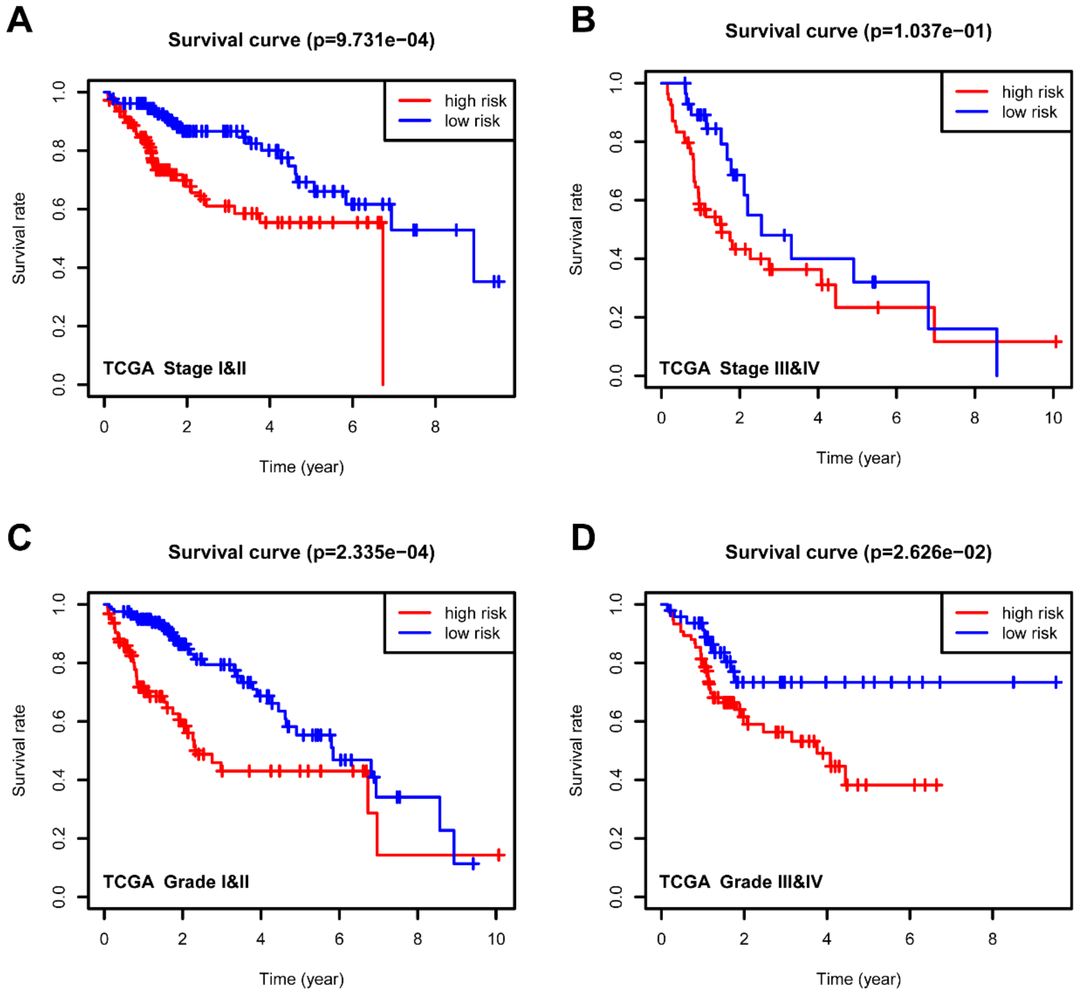

Supplementary Figure 1. The prognostic significance of the signature in sub-groups of TCGA LIHC cohort. (A&B) The Kaplan–Meier curves of overall survival for HCC patients at stage I&II and stage III&IV in TCGA cohort. **(C&D)** The Kaplan–Meier curves of HCC patients at grade I&II and grade III&IV in TCGA cohort. TCGA, The Cancer Genome Atlas.

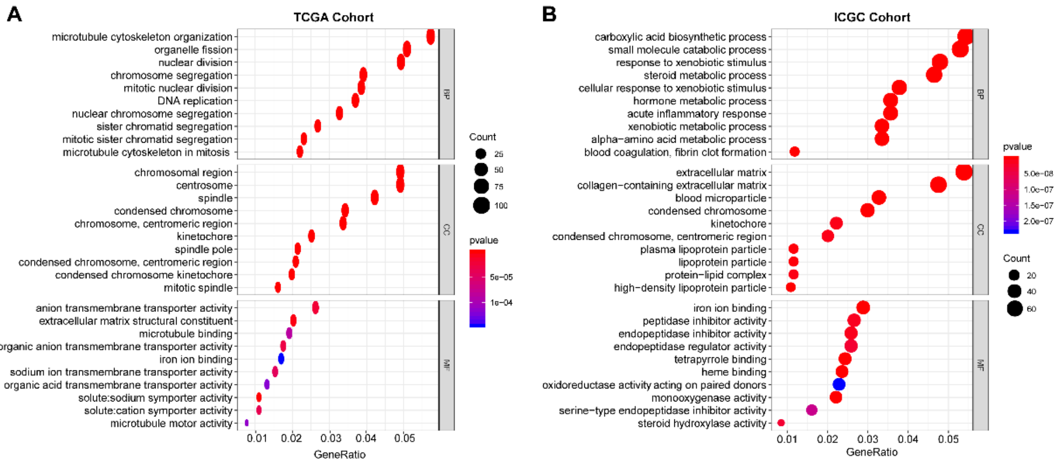

Supplementary Figure 2. The potential functions mediated by the signature. (A) The most significant GO enrichment in the TCGA cohort. **(B)** GO analysis in the ICGC cohort.

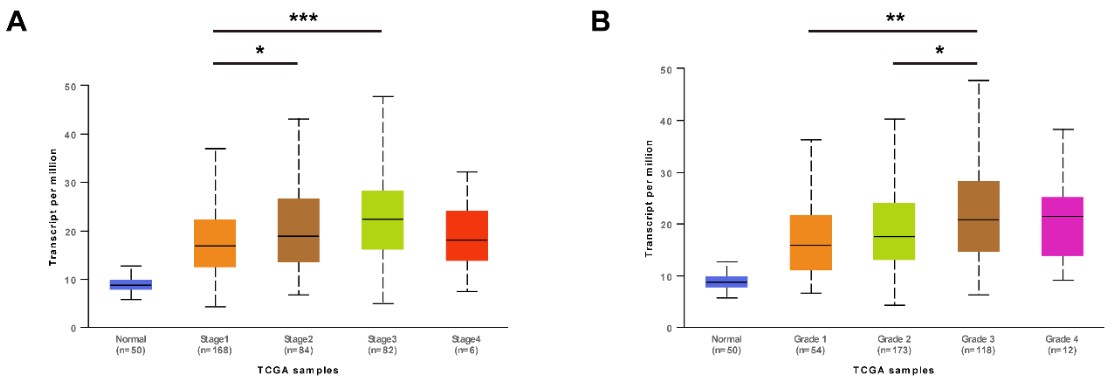

Supplementary Figure 3. The expression features of the 8 risk genes in cases at different stages and grades in TCGA cohort analyzed by Ualcan. (A) The mRNA levels of the 8 USPs in HCC cases at different stages in TCGA cohort. **(B)** The mRNA levels of the 8 USPs in HCC cases at different grades in TCGA cohort. ***, $P < 0.001$; **, $P < 0.01$; *, $P < 0.05$.

A

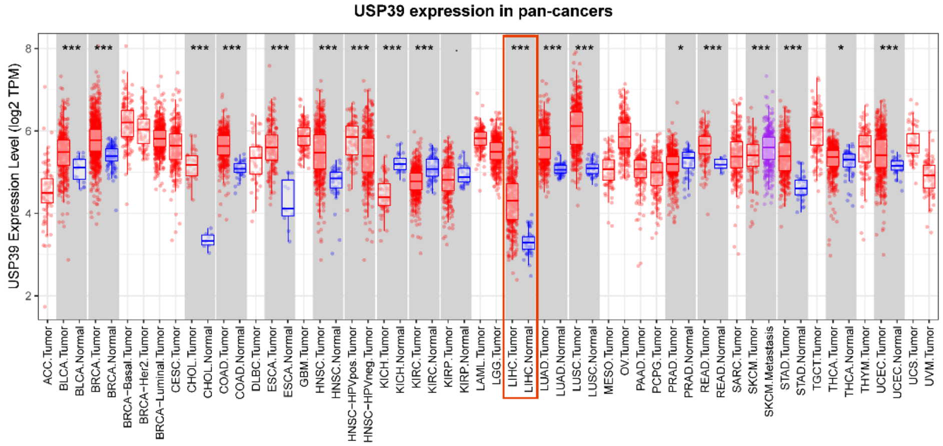

B

Dataset	P-value	Type	Nums	Mean	STD	IQR
GSE22058	2.820e30	HCC	100	10.68	0.3279	0.4036
		Adjacent	97	10.15	0.1462	0.2163
GSE25097	4.650e50	HCC	268	3.107	0.7409	0.8440
		Adjacent	243	2.235	0.3439	0.3500
		Cirrhotic	40	2.221	0.2819	0.4948
		Healthy	6	1.958	0.2944	0.2273
GSE36376	3.730e106	HCC	240	8.184	0.3010	0.3605
		Adjacent	193	7.476	0.1740	0.2264
GSE14520	5.750e53	HCC	225	5.876	0.4920	0.6720
		Adjacent	220	5.152	0.3572	0.4020
GSE46444	0.1102	HCC	88	7.233	1.622	2.586
		Adjacent	48	7.660	1.394	2.417
GSE54236	1.350e10	HCC	81	12.30	0.3059	0.4070
		Adjacent	80	12.01	0.2099	0.2740
GSE63698	1.390e44	HCC	228	7.416	0.4257	0.5293
		Adjacent	168	6.836	0.2969	0.3407
TCGA-LIHC	2.150e30	HCC	351	9.742	0.3973	0.5200
		Adjacent	49	9.264	0.1698	0.2100
GSE64041	0.000007250	HCC	60	7.948	0.3160	0.3625
		Adjacent	60	7.723	0.1902	0.1812
GSE76427	5.190e16	HCC	115	9.103	0.3439	0.4800
		Adjacent	52	8.668	0.2483	0.3050
ICGC-LIRI-JP	4.840e69	HCC	212	3.896	0.4270	0.5500
		Adjacent	177	3.087	0.3005	0.3700

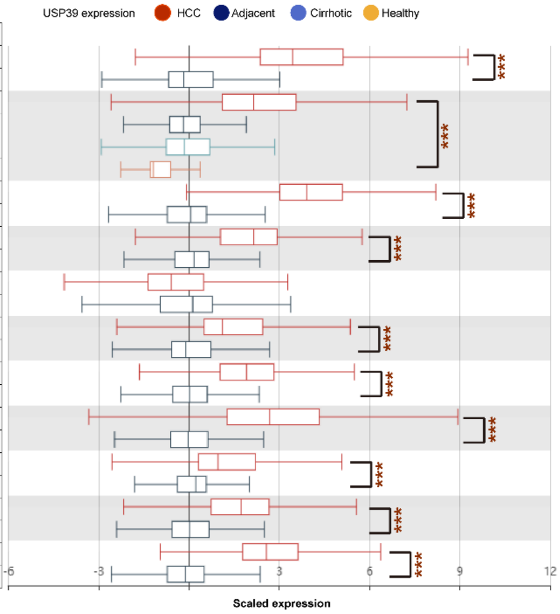

Supplementary Figure 4. The expression features of USP39 in pan-cancers and multiple datasets. (A) The mRNA levels of USP39 in pan-cancers extracted from TIMER database. **(B)** The expression of USP39 in HCC and normal tissues extracted from multiple GSE datasets, TCGA and ICGC(LIRI-JP). ***, $P < 0.001$; **, $P < 0.01$; *, $P < 0.05$.

1.2 Supplementary Table

Table S1. The Univariate analysis of USPs in TCGA

USPs	HR	HR.95L	HR.95H	P-value	USPs	HR	HR.95L	HR.95H	P-value	USPs	HR	HR.95L	HR.95H	P-value
**USP1**	1.105492	1.06508	1.147436	1.30E-07	**USP18**	1.013239	0.936995	1.095686	0.741774	**USP38**	0.991343	0.877897	1.11945	0.88849
**USP2**	0.940448	0.865857	1.021464	0.14532	**USP19**	1.073998	1.018867	1.132112	0.007928	**USP39**	1.132211	1.076745	1.190534	1.26E-06
**USP3**	1.077176	0.955391	1.214484	0.224569	**USP20**	1.027685	0.909124	1.161708	0.66237	**USP40**	1.072905	0.974422	1.181341	0.152
**USP4**	1.066441	0.971569	1.170578	0.175989	**USP21**	1.143389	1.07443	1.216775	2.42E-05	**USP41**	9.397853	0.345777	255.4238	0.183617
**USP5**	1.018311	0.996591	1.040505	0.099038	**USP22**	1.03933	1.019957	1.059071	5.86E-05	**USP42**	1.432602	1.094373	1.875366	0.008889
**USP6**	10.71105	0.014541	7889.978	0.481456	**USP24**	1.212659	1.115389	1.318412	6.19E-06	**USP43**	0.887012	0.715737	1.099274	0.273385
**USP7**	1.038194	0.982238	1.097338	0.184848	**USP25**	1.094275	0.970789	1.233469	0.140294	**USP44**	1.331537	0.402026	4.410145	0.639343
**USP8**	1.086892	0.810753	1.457082	0.577426	**USP26**	19.23077	0.148781	2485.69	0.23331	**USP45**	1.488008	0.816823	2.710708	0.194023
**USP9X**	1.036596	0.995777	1.079087	0.079517	**USP27X**	1.06102	0.977139	1.152102	0.158656	**USP46**	1.934237	1.386059	2.699217	0.000104
**USP9Y**	1.009887	0.70302	1.450702	0.957541	**USP28**	1.230621	1.076395	1.406944	0.002385	**USP47**	1.040719	0.938873	1.153612	0.447517
**USP10**	1.063019	1.007845	1.121215	0.02462	**USP29**	3.07E+64	2.83E+34	3.34E+94	2.58E-05	**USP48**	1.299243	1.150344	1.467415	2.50E-05
**USP11**	1.053646	1.013861	1.094992	0.007792	**USP30**	0.983282	0.892458	1.083349	0.733141	**USP49**	1.912864	0.988092	3.703147	0.054302
**USP12**	1.035089	0.961599	1.114195	0.358703	**USP31**	1.090042	0.843585	1.408503	0.509716	**USP50**	0.226824	0.002278	22.58833	0.527398
**USP13**	1.241295	1.109422	1.388844	0.000162	**USP32**	1.166231	1.062783	1.27975	0.001175	**USP51**	1.193173	0.885535	1.607686	0.245677
**USP14**	1.063089	1.023701	1.103993	0.001493	**USP33**	1.119328	1.048786	1.194615	0.000688	**USP53**	0.882019	0.646632	1.203093	0.428003
**USP15**	1.50355	1.084806	2.083933	0.014337	**USP34**	1.143244	0.968765	1.349147	0.113105	**USP54**	1.178028	1.015841	1.366109	0.030164
**USP16**	1.049376	0.960943	1.145947	0.283278	**USP35**	0.977487	0.8686	1.100024	0.705517	**USPL1**	1.182452	0.975723	1.432982	0.087395
**USP17L1**	8.71E+12	1.50E-14	5.07E+39	0.343339	**USP36**	1.094836	1.006594	1.190814	0.034578	**CYLD**	1.028163	0.86019	1.228938	0.760232
**USP17L2**	2.12E-43	3.61E-93	12442052	0.092849	**USP37**	1.935125	1.297669	2.88572	0.001204	**PAN2**	0.973032	0.905383	1.045737	0.457138

HR, hazard ratio.

Table S2. The GSEA analysis of USPs-based signature in TCGA and ICGC datasets.

TCGA KEGG			ICGC HALLMARK			ICGC KEGG			ICGC HALLMARK		
**Pathway**	**NES**	**P**	**Pathway**	**NES**	**P**	**Pathway**	**NES**	**P**	**Pathway**	**NES**	**P**
Glycosylphosphatidylinositol	1.95	0	Mitotic Spindle	1.98	0	Cell Cycle	2.18	0	G2m Checkpoint	2.22	0
Ubiquitin Mediated Proteolysis	1.92	0	G2m Checkpoint	1.88	0.004	DNA Replication	2.07	0	E2f Targets	2.13	0
Neurotrophin Signaling	1.91	0	E2f Targets	1.83	0.008	Ubiquitin Proteolysis	2.07	0	Mitotic Spindle	2	0
Cell Cycle	1.91	0	DNA Repair	1.77	0.026	Oocyte Meiosis	2.04	0	DNA Repair	1.85	0.004
Oocyte Meiosis	1.9	0.002	Protein Secretion	1.75	0.012	Homologous Recombination	2.03	0	Myc Targets V1	1.8	0.021
Homologous Recombination	1.89	0.004	PI3K Akt Mtor	1.71	0.004	Mismatch Repair	1.99	0	PI3K Akt Mtor	1.76	0.01
Long Term Potentiation	1.86	0.002	Unfolded Protein	1.67	0.008	Adherens Junction	1.95	0	Unfolded Protein	1.7	0.024
Base Excision Repair	1.85	0.004	Wnt Beta Catenin	1.66	0.02	Spliceosome	1.94	0	Protein Secretion	1.65	0.034
Thyroid Cancer	1.85	0.004	MYC Targets V1	1.63	0.047	Water Reabsorption	1.92	0	MYC Targets V2	1.59	0.061
Endometrial Cancer	1.84	0.002	Spermatogenesis	1.6	0.012	Base Excision Repair	1.89	0	Wnt Beta Catenin	1.59	0.036
ERBB Signaling Pathway	1.81	0	TGF Beta Signaling	1.43	0.091	Nucleotide Excision Repair	1.81	0.012	Mtorc1 Signaling	1.47	0.108
Nucleotide Excision Repair	1.81	0.008	Mtorc1 Signaling	1.43	0.105	Colorectal Cancer	1.8	0.002	Notch Signaling	1.46	0.102
Endocytosis	1.79	0	Heme Metabolism	1.41	0.062	Notch Signaling Pathway	1.79	0.002	Spermatogenesis	1.45	0.045
Oocyte Maturation	1.79	0.004	Hedgehog Signaling	1.36	0.133	Glycosylphosphatidylinositol	1.74	0.018	TGF Beta Signaling	1.44	0.107
RNA Degradation	1.77	0	UV Response	1.35	0.137	Phosphate Metabolism	1.72	0.008	UV Response	1.41	0.085
Phosphate Metabolism	1.76	0.002	P53 Pathway	1.34	0.105	Chronic Myeloid Leukemia	1.72	0.008	Apical Junction	1.27	0.189
Notch Signaling Pathway	1.75	0.004	Androgen Response	1.32	0.103	Renal Cell Carcinoma	1.72	0.012	Androgen Response	1.26	0.171
DNA Replication	1.75	0.021	Glycolysis	1.31	0.126	Endocytosis	1.71	0.006	Glycolysis	1.24	0.214
Rig I Like Receptor Signaling	1.75	0.002	Apoptosis	1.3	0.147	Wnt Signaling Pathway	1.7	0.002	Heme Metabolism	1.24	0.161
Chronic Myeloid Leukemia	1.75	0.004	Notch Signaling	1.28	0.184	Autophagy Regulation	1.56	0.047	UV Response	1.22	0.245

NES, Normalized enrichment score.

Table S3. The expression of the USPs in GSE89377.

USPs expression (log2)	Normal	Low dysplastic nodules	High dysplastic nodules	Early HCC	Grade 1 HCC	Grade 2 HCC	Grade 3 HCC
**USP1**	5.815537	5.816198	5.819442	5.75515	5.795782	5.810935	5.806871
**USP13**	8.221288	8.029166	8.268219	8.271848	8.115154	8.240152	8.274836
**USP22**	6.73504	6.931075	6.975362	7.072378	6.828211	6.948086	7.149749
**USP24**	9.20024	8.926327	9.118644	9.021938	9.00174	9.153983	9.191032
**USP29**	5.80225	5.792379	5.772693	5.850815	5.8001	5.800709	5.806731
**USP39**	8.416181	8.447653	8.667896	8.533437	8.760809	9.009042	9.141733
**USP48**	6.93118	6.764356	6.833197	6.668841	6.692701	6.66117	6.817339
**USP54**	6.101342	6.027529	6.0596	5.988791	6.025785	6.145241	6.130518
